# Just kidding: the evolutionary roots of playful teasing

**DOI:** 10.1098/rsbl.2020.0370

**Published:** 2020-09-23

**Authors:** Johanna Eckert, Sasha L. Winkler, Erica A. Cartmill

**Affiliations:** 1 Department of Anthropology, University of California Los Angeles, Haines Hall, Los Angeles, CA 90095, USA; 2 Department of Comparative Cultural Psychology, Max Planck Institute for Evolutionary Anthropology, Deutscher Platz 6, 04103 Leipzig, Germany; 3 Department of Psychology, University of California Los Angeles, Franz Hall, Los Angeles, CA 90095, USA

**Keywords:** social cognition, theory of mind, play, humour, non-human primate, great ape

## Abstract

Accounts of teasing have a long history in psychological and sociological research, yet teasing itself is vastly underdeveloped as a topic of study. As a phenomenon that moves along the border between aggression and play, teasing presents an opportunity to investigate key foundations of social and mental life. Developmental studies suggest that preverbal human infants already playfully tease their parents by performing ‘the unexpected,’ apparently deliberately violating the recipient's expectations to create a shared humorous experience. Teasing behaviour may be phylogenetically old and perhaps an evolutionary precursor to joking. In this review, we present preliminary evidence suggesting that non-human primates also exhibit playful teasing. In particular, we argue that great apes display three types of playful teasing described in preverbal human infants: teasing with offer and withdrawal, provocative non-compliance and disrupting others' activities. We highlight the potential of this behaviour to provide a window into complex socio-cognitive processes such as attribution of others’ expectations and, finally, we propose directions for future research and call for systematic studies of teasing behaviour in non-human primates.

## 1. Introduction

‘You can't tease other people unless you can correctly guess what is in their minds and make them suffer or laugh because of your knowing.’ – Daniel N. Stern [1]

We are all familiar with teasing. Be it as teaser, recipient or observer, from early childhood onwards, everyone experiences this hard-to-define phenomenon that occurs in social interactions all around the world [2]. Its inherent dichotomy—the mix between aggressive and playful elements—can affect the relationship between teaser and recipient in contrasting ways [3–9]. If the aggressive component predominates, teasing may be perceived as more hostile, or even as bullying, and has the power to result in serious harm and damage a relationship permanently. If teasing is more playful and humorous, the teasing event may be mutually enjoyable for both teaser and recipient, and potentially lead to greater closeness (e.g. [10,11]). Accordingly, the proposed functions of teasing are highly diverse and range from gaining social status to enforcing social norms, resolving conflicts and enhancing interpersonal relationships [2–12].

From a psychological perspective, playful teasing^1^, i.e. behaviour that sits on the playful, non-aggressive end of the teasing spectrum, is particularly interesting for two reasons. First, in contrast with other, more obviously aggressive forms of teasing, playful teasing is highly ambiguous. Thus, it most likely involves ‘mind-reading’ skills on both the side of the teaser and the recipient. For playful teasing to be successfully interpreted as affiliative rather than aggressive, the teaser, to some extent, has to understand the recipient's expectations and predict their likely reaction. Likewise, the recipient needs to draw accurate inferences and correctly identify the teaser's intent as affiliative, looking beyond any mildly abrasive behavioural elements. In line with the hypothesis that playful teasing is a cognitively complex form of teasing, studies have shown that teasing is viewed as a potentially positive social interaction only by older children and adolescents. Younger children, by contrast, recognized only the negative sides of teasing [9,12,13]. The second reason playful teasing is interesting psychologically is that it has potential to create mutual amusement. A shared humorous experience is an interaction of positive affective valence and may strengthen social bonds [14–16]. Hence, playful teasing is noteworthy because of its implications for higher socio-cognitive abilities, as well as its potential relevance to the origins and functions of humour.

## 2. Playful teasing in human infants

In contrast with studies showing that only older children interpret teasing behaviour as positive, research on preverbal infants suggests that some forms of non-verbal playful teasing appear before a child's first birthday. Reddy & coworkers [17–21] conducted a series of observational and interview studies and found evidence for positive teasing behaviour in infants as young as 8 months. They described three types of playful teasing in infants: offer and withdrawal of objects or the self (e.g. offering the parent an object and quickly pulling back as the parent reaches for it), provocative non-compliance (e.g. attempting to perform a prohibited action or refusing to perform an expected behaviour) and disrupting others' activities (e.g. taking objects from others when they engage with them; also see, e.g. [22–24] for similar findings in toddlers). Typically, infants repeated these acts several times, all while looking and smiling at the recipient, waiting for an emotional reaction. Infants seemed to seek positive reactions; acts that led to distress in the recipient were rarely repeated [18]. In these exchanges, which typically occurred in moments of neutrality or boredom [21], infants appeared to use teasing to explore limits of newly acquired skills or social agreements, as well as to invite and maintain playful and mirthful interactions [21,23–25].

What these types of infant teasing have in common, and what differentiates them from other types of play initiation, is that the teaser performs an unexpected act, apparently deliberately violating the recipient's expectations, mutual understandings or shared conventions in order to provoke a reaction [21,24–27]. From a socio-cognitive point of view, these behaviours are particularly intriguing, because the ability to manipulate others’ expectations presumably requires relatively sophisticated inferences regarding others' actions and mental states [18,25,26]. For instance, in an offer-withdrawal event, the infant seems not only to anticipate that the recipient will reach for an offered object, but also that she will react with surprise if the offer is withdrawn. The infant seems to be aware of a set of behavioural norms and anticipates what actions would violate those norms and, thus, also violate the recipient's expectations. The infant, therefore, seems to actively create expectations in the other in order to playfully disrupt them. Structurally, this sequence resembles a simple joke, with a familiar setup (the offer) and a surprising punch line (the withdrawal). Like most jokes, playful teasing appears enjoyable for both parties. It relies on an understanding of the familiar event's structure and an appreciation of the incongruous nature of the punch line (e.g. [28,29]). Early forms of joking behaviour in infants described in other studies [26,27,30] also involve playing with rules and expectations (e.g. putting inappropriate objects, such as sponges, in their mouths, while laughing and looking for a reaction [27]).

Importantly, infants can also be knowing recipients of playful teasing and react with laughter when parents do absurd things in an affiliative context, such as drinking from the infant's milk bottle [21,24,31]. Again, what infants seem to find amusing in others' behaviour reveals their awareness of expected (and unexpected) ways to behave. Playful teasing involving the violation of other's expectations might be an important developmental marker of the awareness of other minds and behavioural norms and might represent one of the earliest forms of humour.

The occurrence of playful teasing in preverbal human infants suggests that language is not a prerequisite for this type of behaviour and, thus, opens up intriguing questions about the evolutionary roots of this multifaceted phenomenon. Is playful teasing an early developmental indicator of humans’ unique socio-cognitive skills? Or is it an evolutionarily old behaviour that we might share with other animals, most notably our closest living relatives, the non-human great apes? Answering these questions will help us to develop a better understanding of apes' socio-cognitive capacities and give us intriguing insights into the phylogenetic origins of humour.

## 3. Teasing in non-human primates

Teasing in non-human animals is drastically understudied and has mainly focused on aggressive behaviours (sometimes described as harassment or quasi-aggression). The earliest mention of aggressive teasing in the primate literature stems from Wolfgang Köhler's observations of captive chimpanzees in 1927 ([32], table 1). Half a century later, de Waal and Hoekstra described aggressive teasing in juvenile chimpanzees: ‘they approached quietly-sitting apes, threw sand or sticks towards them, stamped with their feet on the ground, and ran away if their object jumped to its feet, but shortly afterwards came back to throw sand again, and so on. Especially in senior females, this teasing provoked aggressive reactions’ [41].

**Table 1.** Examples of teasing behaviour from the literature on non-human primates.

year	author	ref	species	aggression or play	description of behaviour
1927	Köhler	[32]	chimpanzee (*Pan troglodytes*)	A/P	describes playful teasing with sticks and other objects between chimps, which may result in aggression. Also describes chimps teasing humans or birds by startling them or with object offer-withdrawal
1940	Maslow	[33]	chimpanzee (*Pan troglodytes*)	A/P	discusses rough play and teasing as a form of aggression between close individuals with disparate rank
1945	Hebb	[34]	chimpanzee (*Pan troglodytes*)	A	describes teasing via spitting water at or startling the recipients, who responded with ‘anger’ and aggression
1967	Rowell	[35]	olive baboon (*Papio anubis*)	A	aggressive teasing/tormenting of females by young males, more often in captivity than in the wild; ‘approach-retreat’ behaviour; chasing, mouthing, pulling fur, poking. Recipients reacted with avoidance or submission
1968	van Lawick-Goodall	[36]	chimpanzee (*Pan troglodytes*)	P	‘pestering’ of adults by infants leaping onto them, biting or pulling their hair, hitting them or dangling above and kicking at them; tolerated and sometimes resulting in play
1972	Dolhinow	[37]	grey langur (*Presbytis entellus*)	A	harassment of adult males by juveniles in affiliative or mating contexts, accompanied by ‘squealing,’ sometimes tolerated. Described as a possible example of teasing in Adang [38]
1977	de Waal	[39]	long-tailed macaque (*Macaca fascicularis*)	A	juveniles pulling the tail of the alpha male or sitting/hanging in front of him and waving their arms; sometimes led to aggression
1978	Bramblett	[40]	vervet monkey (*Chlorocebus pygerythrus*)	P	description of a juvenile playfully pulling the tail of the alpha female, then running away from her aggressive response
1980	de Waal & Hoekstra	[41]	chimpanzee (*Pan troglodytes*)	A	‘annoying’ behaviours such as throwing sand or sticks, jumping on another's head, or other ‘presumably discomforting actions,’ sometimes leading to aggression
1982	Boggess	[42]	grey langur (*Presbytis entellus*)	A	juvenile males teasing adult males by circling them and sometimes slapping them before rapidly withdrawing
1984; 1985; 1986	Adang	[38,43,44]	chimpanzee (*Pan troglodytes*)	A	discusses teasing as exploratory aggression, explicitly excluded playful behaviours
1985	Kummer & Goodall	[45]	chimpanzee (*Pan troglodytes*)	A/P	describes frequent adolescent male ‘challenging’ of adult females with aggressive displays, with one incident resulting in the female tickling the displaying male and the male producing play-specific vocalizations
1986	Goodall	[57]	chimpanzee (*Pan troglodytes*)	A	description of 3- to 5-year-old juveniles dangling above resting adults and kicking at their head and shoulders, sometimes resulting in aggression
1986	Hiller & Patterson	[46]	western lowland gorilla (*Gorilla gorilla*)	P	sign-language-trained gorilla Koko answered questions with obviously wrong answers while displaying a play-face
1990	Pusey	[47]	chimpanzee (*Pan troglodytes*)	A/P	‘challenging’ of adult females by younger males; often seemed playful, but sometimes occurred with piloerect hair or contact aggression
1991	Patterson & Linden	[48]	western lowland gorilla (*Gorilla gorilla*)	P	sign-language-trained gorilla Koko produced notably altered signs for familiar words while displaying a play-face
1995	Mendoza-Granados & Sommer	[49]	chimpanzee (*Pan troglodytes*)	P	defined ‘para-play’ as behaviour that appeared playful but involved strong agonistic components, drawing on Adang's definition of teasing
1996	Butovskaya & Kozintev	[50]	chimpanzee (*Pan troglodytes*) and orangutan (*Pongo pygmaeus*)	A/P	teasing of both humans and conspecifics by apes by throwing feces and other objects. Described as quasi-aggression or mock aggression, but accompanied by a play-face. Relevance for the origins of schadenfreude and humour are discussed
1996	de Waal	[51]	chimpanzee (*Pan troglodytes*)	A/P	teasing as way to gather information about the social environment and to investigate authority. Continuum from playful teasing to aggressive teasing
1999	Nishida	[52]	chimpanzee (*Pan troglodytes*)	A	harassment of adult females by young males; anecdote of adolescent female ‘trifling’ with a young male, perhaps playfully
2003	Nishida	[53]	chimpanzee (*Pan troglodytes*)	A	harassment of adult females by young males in order to improve rank. Use of objects (e.g. branches) described as common
2007	Call & Tomasello	[54]	chimpanzee (*Pan troglodytes*)	P	teasing with offer-withdrawal of objects or limbs
2010	Cartmill & Byrne	[55]	orangutan (*Pongo pygmaeus*)	P	adult female observed in a playful teasing interaction with her juvenile daughter, using the ‘fake’ gesture
2018	Krupenye *et al*.	[56]	bonobo (*Pan paniscus*)	P	offer-and-withdrawal of sticks toward human experimenters

Teasing was systematically studied by Adang in his long-term observational study of young chimpanzees (1.5–7.5 years-of-age) in Arnhem Zoo [38,43,44]. Because he was interested in ‘quasi-aggressive’ behaviours, Adang focused on agonistic forms of teasing, such as ‘bluff-like’ behaviours (e.g. stamping), swinging or throwing of objects, and hitting or kicking. He reported that such teasing behaviours typically occurred during vigorous social activity (play or conflict) and were mostly directed at adults outside the teaser's sub-group [38]. The reactions of the targeted individuals were variable, ranging from ignoring to aggression, submission, flight or affiliation, and negative responses appeared to reinforce the teasing behaviour [38]. Adang theorized that juveniles used teasing to learn about or to establish dominance relationships (a function of teasing that has also been proposed for third- to sixth-grade human children [9]). Similar forms of aggressive teasing have also been observed in wild chimpanzee populations [47,52,53,57] and in other primate species (e.g. langurs: [37,42]; macaques: [39], baboons: [35]; table 1 for details^2^).

As in humans, teasing in great apes is highly variable and includes behaviours on a continuum between aggression and play. However, to date, there are no systematic studies on the more playful forms of teasing in non-human primates. This is surprising considering the wealth of research studying play, and in particular play fighting, in non-human primates and other animals (e.g. [58–61]). We believe that the dearth of playful teasing descriptions in the literature stems from a bias in observation: Adang [38,43,44] explicitly excluded all behaviours that were accompanied by a relaxed open-mouth display (a play-specific signal in many primates, also called ‘play-face’). Thus, his studies only captured more agonistic forms of teasing, disregarding acts that were likely to be performed in a positive affective state. Also, several reports of teasing in non-human primates appeared in studies of aggression, naturally biasing the observations towards agonistic forms (e.g. [41]). While studies of teasing are few in number and biased towards aggression, descriptions of behaviour matching the three forms of playful teasing Reddy & coworkers report for human infants (offer and withdrawal, provocative non-compliance and disruption of others' activities) can be found in the ethological and behavioural literature on non-human primates.

### (a) Offer and withdrawal of objects or the self

**Figure 1.**
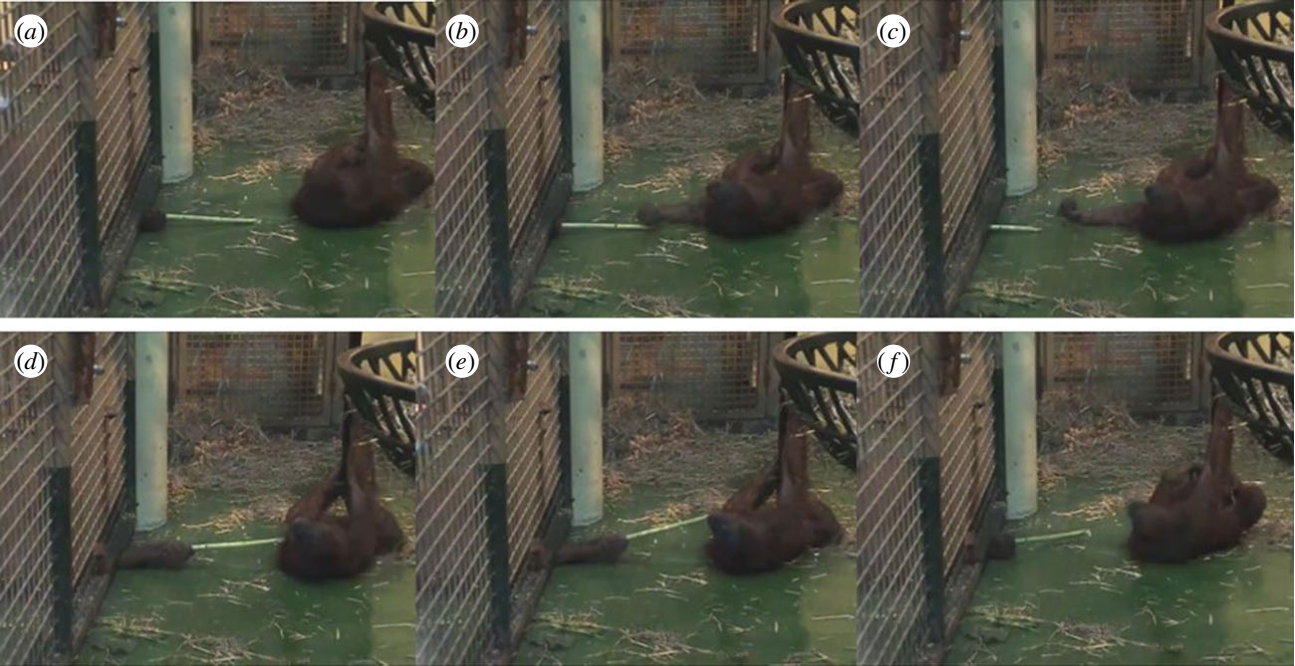
Example of playful teasing with object offer and withdrawal. Male orangutan (behind the mesh on the left side) extends a stick towards the female (*a*). As she tries to grasp it (*b*), he quickly withdraws it out of her reach (*c*). Soon after, he extends the stick again (*d*), this time wiggling it close to her face. As soon as she tries to bite the stick (*e*), he retracts it (*f*).

Offer and withdrawal of objects has been described in gestural studies and object transfer studies for several great ape species. Call & Tomasello [54] described a visual gesture displayed by chimpanzees at Yerkes Primate Research Center in Lawrenceville, GA, which they called ‘ball offer:’ the signaller ‘present[s] ball to the recipient and take[s] it back when recipient approaches.’ These offer-withdrawals, which also occurred with other objects or limbs, were recorded in the context of play and, thus, were likely not aggressive (J Call 3 March 2019, personal communication). Cartmill & Byrne [55] described a ‘fake’ gesture used by an orangutan at Twycross Zoo in which the arm was quickly extended towards the recipient and then retracted. The authors report it was used by an adult female ‘during a particular teasing exchange with her juvenile daughter.’ Again, this gesture was interpreted as affiliative and playful. New analysis of this video corpus [55] revealed additional instances of offer-withdrawal in orangutans of different ages involving both objects (e.g. sticks; figure 1) and limbs (e.g. hands).

A recent experimental study [56] described offer and withdrawal exchanges in bonobos (*Pan paniscus*). This experiment was designed to test whether bonobos would retrieve and transfer an object (a stick) to help a human experimenter. Interestingly, subjects often retrieved the object but did not transfer it. Instead, several individuals repeatedly ‘…responded with what appeared to be teasing instead of helping (i.e. gesturing toward E1 with stick in hand, often moving the stick close and then pulling it back, and ultimately refusing to transfer the stick)’ [56]. The authors interpreted this behaviour as an attempt to initiate positive social interaction. Köhler [32] described a similar case of interspecies object offer-withdrawal: ‘a chimpanzee will sometimes hold his slice [of bread] between the meshes of wire; a hen approaches to peck at the bread, but before she can do so, it is pulled back again. At one meal this joke will be repeated about fifty times.’

These studies [32,54–56], together with our own observations, suggest that chimpanzees, bonobos and orangutans all engage in the offer and withdrawal of objects or the self, as described in human infants. An alternative explanation is that the apes did not intend to tease, but rather began to transfer the object and then changed their minds. However, this seems unlikely given that apes, like infants, often repeated the behaviour several times, and the context was typically relaxed and playful (e.g. [32,56]; personal observations). To verify the interpretation of this behaviour as a form of play, future research should explore whether apes, like infants, often produce play signals during offer-withdrawal events.

### (b) Provocative non-compliance

Detecting instances of provocative non-compliance is challenging because it is difficult to discriminate intentional non-compliance from the inability to comply and identify the motivation for non-compliance as stemming from provocation, rather than idleness. An example that is probably familiar to researchers doing experimental research with apes is a subject who refuses to give back an object. This could be an example of provocative non-compliance, meant to provoke a reaction in the experimenter and perhaps to initiate social interaction. However, it is similarly possible that the ape does not understand the command ‘give back’ and therefore is unable to comply. Also, the ape might know the command but simply wants to keep the object for herself, irrespective of the experimenter's reaction. In order to identify potential provocative non-compliance, it is essential to consider whether the individual is capable of responding to the request and whether it directs its attention at the presumed recipient of the provocation (e.g. the experimenter), rather than at the object itself.

Descriptions of this behaviour in non-human species are scarce. To our knowledge, the only explicit mentions in the literature come from studies of language-trained apes. For example, the sign-language-trained western lowland gorilla Koko regularly gave incorrect answers to questions when her caretakers were confident she knew the right answer. In one occasion, Koko answered the question ‘What does Penny use to clean your teeth?’ by signing ‘foot,’ and the question ‘What does Penny put on your toothbrush?’ by signing ‘nose,’ before lifting her foot to her nose and showing a play-face [46]. In other instances, Koko refused to produce signs for words she previously mastered, only to produce a notably altered version eventually (e.g. executing the sign for ‘drink’ to the ear instead of the mouth), again while exhibiting a play-face ([48]; see [62,63] for more examples of seemingly playful mislabelling of objects in language-trained apes). However, we should exercise some caution when interpreting these anecdotes. First, it is unclear what training history preceded these seemingly humorous scenes. Second, a difficulty in studies of sign-language-trained apes is subjectivity in interpreting the apes' (often very ambiguous) signs, and the consequent risk of over-interpretation and anthropomorphism. Therefore, while these reports do suggest that great apes might exhibit forms of provocative non-compliance similar to those found in human infants, these preliminary findings need to be confirmed through empirical research.

### (c) Disrupting others’ activities

Identifying when an individual purposefully disrupts others' activities for the purposes of affiliative provocation is not a trivial task. Behaviour disruptions can have multiple causes and motivations. While some might be intended to get a playful and perhaps surprised response from the recipient, others may aim to achieve a different goal (e.g. purposely disrupting a resting individual in order to start moving together or accidentally disrupting another's activity because your path crosses theirs). As with other types of playful teasing, the co-occurrence of play-specific signals would provide more information about motivation or affective state of participants.

Reports of wild apes describe instances of playful disruptions. Van Lawick-Goodall [36] writes that chimpanzee infants ‘were often seen pestering older individuals who were peacefully resting or grooming: the infants leaped onto them, biting or pulling their hair, hitting them or dangling above and kicking at them. Such behaviour was invariably tolerated—the adults concerned either began to play, actively, or merely reached out and pushed the infant to and fro as it dangled.’ An inspection of the video corpus collected for Cartmill & Byrne [55] revealed several instances of orangutans seemingly engaging in playful disruption of others' activities. For example, a juvenile male at Twycross Zoo repeatedly approached his mother and sister, who were grooming each other, from behind. He then briefly poked one of them, or pulled their hair, before withdrawing. He repeated this until one of the two followed him and engaged in rough-and-tumble play. Other papers have mentioned similar types of teasing in great apes (e.g. [41]), but described the behaviour as ‘annoyance’ rather than play.

## 4. Cognitive implications of playful teasing

Some authors have proposed that playful teasing in human infants provides a window into their rich early ‘theory of mind’ abilities, as well as into proto-forms of humour (e.g. [18,21,23–27,64]). If apes (or other animals) engage in similar forms of playful teasing, do they also have some understanding of the expectations of others? Is it possible that great apes, like human infants, deliberately play with these expectations for the sake of amusement?

The study of ‘theory of mind,’ i.e. the ability to ascribe mental states to others, has been of central interest in comparative psychology for several decades (see [65–67] for reviews). There is ample evidence showing that great apes (i) ascribe *intentions and goals* to others (e.g. [66,68–70]), (ii) are aware of *attentional states* of others (i.e. what they can see or hear; e.g. [71–74]) and (iii) *make use of this knowledge* in both competitive and cooperative contexts (e.g. [75]). Crucially, recent research demonstrated that apes are also capable of ‘mind-reading’ abilities that require a simultaneous representation of two conflicting views of the world: one's own (correct) perspective and the (incorrect) perspective of another individual [76]. Hence, great apes are not only sensitive to what other individuals intend to do and what they know, but they also have some understanding of others' beliefs, even when these beliefs conflict with reality (also see [77–79] for similar findings on false belief attribution in young children).

Playful teasing events, such as the offer-withdrawal, presumably involve rich inferences on both the side of the teaser and the side of the recipient. A typical offer-withdrawal event starts with the teaser making an ‘offer’ gesture, inviting the recipient to reach for an extended object or limb. All species of great apes produce offer gestures, e.g. in the context of food sharing [80] or grooming [81]. Hence, it is reasonable to assume that both parties are aware of the typical use of this gesture to draw attention to a body part of the signaller or to transfer something to the recipient. Also, there is evidence that apes produce this gesture type *intentionally* to pursue a particular goal [54,82]. Gestures are typically deemed to be intentional if they are (i) motorically ineffective, (ii) directed towards another individual, (iii) goal-directed and (iv) demonstrate flexibility in their usage. Goal-directedness is often shown through the use of response waiting or through persistent attempts to communicate. Teasing events may take different forms than gestures, but if they are fundamentally communicative in nature and are aimed at eliciting a particular response from the target, they will likely demonstrate the same markers of intentionality as seen in ape gesturing.

These markers seemed to be present in the videos of orangutan offer-and-withdrawal events (collected for [55]). The teaser usually seemed to await a particular response from the recipient after offering the object or limb (anticipating a reaching-out-to-take action). If this response was not given, the teaser slightly modified or intensified the offer gesture. In one case, an orangutan offered another a stick by holding it within their reach. When the recipient did not reach for it (because it had previously made an unsuccessful attempt to obtain the stick), the teaser started waving the object in front of the recipient's face. Only once the recipient reached for the stick, did the teaser withdraw the offer (figure 1*d–f*). While more systematic observations of object-teasing are needed, this behavioural sequence (waiting for a response and modifying the signal when the response did not occur) suggests that apes produced this offer gesture intentionally to elicit the other's attempt to retrieve the item, a response which they then thwarted by withdrawing the offer. It is undeniably difficult to attribute specific goals to teasers (or gesturers) without relying solely on the intuition of the observer. However, careful examination of the satisfying conditions under which the teaser (or gesturer) stops acting can be used to test the observer's attributions of the goal. This has been a very successful method for analysing the meanings of ape gestures (e.g. [55]). Once the offer is withdrawn, the recipient needs to interpret the intention of the teaser as being affiliative (or neutral) rather than aggressive. Primates typically respond with anger when humans retract offers (e.g. [83]). In order to maintain a positive interaction surrounding the teasing behaviour, recipients cannot rely on the teasing behaviour alone but must take into account their relationship with the teaser, the teaser's affective state and other contextual information. These cognitive inferences are even more critical in the absence of overt play signals (e.g. play-face). Because teasing can be a highly ambiguous behaviour, responding to teasing as play—especially participating in teasing ‘games’ like repeated offers with withdrawal—requires careful assessment of social cues and relationships as well as inferences about the other's motivation in a given interaction (also see [58] for a valuable discussion on how different animal species manage and overcome the ambiguity of actions during play fighting).

## 5. Humorous play with others' minds?

An intriguing question is whether ape teasers not only expect a specific action response from the recipient but whether they also attribute *expectations* to their recipient (e.g. the expectation that the teaser will transfer an object). As mentioned above, (false) belief attribution has only been demonstrated recently in apes in a single study employing implicit measures [76]. The occurrence of teasing with offer and withdrawal could provide a hint that apes not only have an implicit understanding of others’ beliefs but that they may even actively create false beliefs by intentionally evoking expectations in the other, before disrupting them.

The deliberate creation of false expectations has previously been discussed in the context of a structurally similar but functionally different behaviour displayed by non-human primates: tactical deception (see [84,85] for reviews). Tactical deception describes ‘acts from the normal repertoire of the agent, deployed such that another individual is likely to misinterpret what the acts signify, to the advantage of the agent’ [81]. There is evidence that great apes use tactical deception in naturally occurring situations [85] and experimental contexts (e.g. [86,87]). Hence, apes do occasionally use false communicative signals to influence the behaviour of others.

One idiosyncrasy of playful teasing is that, in contrast with tactical deception, no immediate fitness benefits are apparent. One possible explanation is that playful teasing constitutes a safe domain within which to explore social rules and boundaries (see [21,23–25]). Research on play fighting in apes suggests that individuals can test social rules in play that they might not be able to explore outside the play context (e.g. [88]). Another possibility is that the teasing behaviour evokes a positive affective state in the teaser and perhaps also in the recipient. For human infants, a suggested proximate function of playful teasing is to create a shared humorous experience between teaser and recipient (e.g. [21]), which may strengthen their social bond (but see [27] for an alternative proposition). Social bonds are critically important for fitness in non-human primates [89,90]. Is it, thus, possible that apes also experience positive emotions such as amusement when playfully teasing others and that sharing such moments enhances bonding between individuals?

This question is related to a more general discussion about whether great apes, or any non-human animals, appreciate humour. One widely used definition of humour states that incongruity with respect to reality is the source of humour [28,29]. This incongruity must be in the form of a benign (i.e. harmless) expectation violation; otherwise, it will elicit negative emotions instead of amusement [91]. Creating this incongruity involves a cognitive understanding of action norms and how those can be violated [26]. Great apes have previously demonstrated such understanding in the context of imitation recognition [92,93]. Moreover, apes' playful teasing fulfils the criteria of the benign expectation violation theory [91]. Hence, technically, playful teasing might be viewed as a humorous act. The question is whether apes, like humans, also appreciate this humorous component and experience a positive emotional state during teasing interactions.

In humans, studying humour and its effects on affective states is eased by the fact that, from early infancy onwards, amusement is often (but not always) accompanied by a distinct emotional expression: laughter [94,95]. Importantly, great apes also emit laughter-like vocalizations (though mostly during dynamic social activities like wrestling, tickling and chasing games [96–98]), suggesting that apes may experience joy during social interactions. Chimpanzees not only laugh spontaneously but also after hearing the laughter of others [99]. Chimpanzee play sessions involving laughter contagion last longer than play involving only spontaneous laughter (or no laughter at all), suggesting that, like in humans, shared laughter may facilitate positive social interaction and enhance bonding (also see [100,101] for evidence of contagious play vocalizations in rats and kea parrots).

Studies documenting offer-withdrawal, provocative non-compliance or disruption of other's activities in apes reported that these behaviours occurred in playful contexts and, thus, likely involved a positive emotional state. However, most studies did not report on any affective signals, such as play-face or laughter (but see [48,50]). Hence, while teasing constitutes an excellent place to look for potential antecedents of joking behaviour and humour in great apes, future research will need to pay close attention to markers of positive affect during these activities. Finding evidence that both teaser and recipient exhibit positive affective states would strengthen the hypothesis that non-human animals are capable of creating and appreciating humorous experiences, and that they, like human infants, use mild expectation-violations to strengthen their bonds.

## 6. Need for systematic study

Despite its enormous potential, teasing remains dramatically understudied. We need systematic observational studies of teasing in order to understand the similarities and differences between human and non-human forms of teasing, and to reconstruct the evolutionary roots of this intriguing behaviour.

Due to the lack of documented responses to playful teasing events in the animal literature, our review has mainly focused on the teaser and the type of inferences the teaser likely makes (following work on teasing in human infants). However, the inferences made by the recipient of a teasing event are of equal interest and should be a focus of future research. Paying close attention to the triadic relationship between teaser, recipient, and teasing behaviour, as well as to which factors within this relationship determine the outcome of a teasing event, will be crucial in categorizing and defining different forms of teasing accurately. Answering questions about the relationship between teaser and recipient (e.g. closely or loosely bonded individuals?), the age of teasers (e.g. does the incidence or form of teasing change over development?), their dominance ranks (e.g. are teaser and recipient close in rank or is one role typically higher ranking?) and the contexts in which teasing occurs (e.g. in moments of neutrality/boredom or during vigorous social activity?) will aid in determining different types and potential functions of teasing. Particular attention should be paid to the affective states of both parties (e.g. as expressed through play signals such as play-face or laughter) and to the presence of behaviour repetitions and role reversals. If ape teasing is indeed a proto-form of joking, serving to enhance the bond between teaser and recipient, we might find markers of positive affect, observe more positive (i.e. playful) than negative (i.e. aggressive) outcomes and find this behaviour most often exhibited between closely bonded individuals. Pinning down the functions of non-human primate teasing will further inform our knowledge of how and why this ubiquitous behaviour evolved in humans. In addition to systematic observational research, experimental studies can address critical open questions regarding the underlying cognitive abilities of playful teasing, and their implications for the origins of humour. Specifically, experimental studies will be essential to investigate whether apes indeed attribute expectations to others and whether they enjoy watching benign violations of others’ expectations.

This review has focused on humans' closest living relatives, the great apes. However, it is certainly possible that other, more distantly related species also exhibit cognitively rich forms of playful teasing. Many animal species engage in play fighting, a behaviour that resembles teasing in that it constitutes a blend of both competition and cooperation, which sometimes involves ambiguous behavioural elements that can be used to test the boundaries of relationships with others [58,102–105]. Indeed, some researchers have highlighted the structural parallels between play fighting and human verbal play [106]. Also, games like ‘keep-away’, often displayed by dogs, share some structural similarity with offer-withdrawal events [107]. Several anecdotes in the animal behaviour literature describe instances of interspecific teasing, both in primate and non-primate species (see, e.g. [32,59,108]). These events are more likely to be unidirectional, i.e. not mutually enjoyable for both teaser and target. In fact, severe imbalances in power between teaser and target may even result in serious harm to the victim [59].

While it seems plausible that the evolutionary predecessors of playful teasing could be found in other types of play, such as play fighting (also see [109] and [110]), further research is needed to explore whether these behaviours truly resemble the sorts of playful teasing behaviour seen in great apes, involving the active creation and disruption of other' expectations for mutual enjoyment. If playful teasing necessarily involves the manipulation of others' expectations, then it is most likely to be observed in species with complex socio-cognitive abilities. Recent research suggested that monkeys can attribute false beliefs to others [111], and several corvid species have demonstrated sophisticated theory of mind skills [67,112]. These species might, therefore, also have the ability to ‘play with other minds’ through playful teasing. Systematic comparative studies across species will help us reconstruct the evolutionary roots of playful teasing and shed more light on the cognitive prerequisites of this behaviour.

## 7. Conclusion

Teasing presents an intriguing opportunity to investigate key components of social and mental life. The occurrence of playful teasing in preverbal human infants indicates that this behaviour does not rely on symbolic language and may be evolutionary old. We argue that the paucity of playful teasing in the non-human primate literature does not stem from an absence of such behaviours, but rather from a lack of systematic study. In this paper, we have collected preliminary evidence suggesting that great apes may playfully tease others in ways similar to human infants. Further observational and experimental research on playful teasing in primates provides a unique opportunity to study potentially humorous behaviour in non-human species. It can also build upon the existing research demonstrating implicit false belief understanding to strengthen the case for a more sophisticated theory of mind abilities in apes than was previously assumed. Therefore, studying playful teasing in our closest living relatives not only gives us new insights into the phylogenetic roots and potential functions of human teasing behaviour, but might also offer a critical window into the evolutionary origins of our sophisticated socio-cognitive skills. We hope that this article will be a first step in stimulating further research on this intriguing but vastly understudied phenomenon.

## Endnotes

1 We categorize teasing as playful when it is both intended to provoke a positive interaction and is also perceived as such by the recipient. To properly classify teasing as playful, it is, thus, important to take into account both the teaser's and the recipient́s behaviour. Playful teasing attempts can also be unsuccessful, e.g. when the positive intent of the teaser is misunderstood by the recipient. The intended outcome of playful teasing can be play, but also simply attention or other types of (not negative) interactions between teaser and recipient.

2 Please note that this list of studies is most likely not exhaustive. While few studies report on teasing directly, there are likely to be descriptions of similar actions in studies involving ethograms that include social behaviour.

## Data accessibility

This article has no additional data.
